# TRIDENT: inTRaoperative Imaging DEvice based on endogeNous opTical properties to evaluate bowel oxygenation in left-sided colorectal resections. Observational first-in-human pilot safety trial

**DOI:** 10.1007/s00464-026-13429-6

**Published:** 2026-10-12

**Authors:** Alfonso Lapergola, Luca Baratelli, Gabriel Szydlo Shein, Guillaume Philouze, Sylvain Kpekou, Arturo Pardo, Frédéric Triponez, Didier Mutter, Sylvain Gioux, Michele Diana

**Affiliations:** 1 Department of General, Digestive, and Endocrine Surgery, University Hospital of Strasbourg, Strasbourg, France; 2 IHU-Strasbourg, Institute of Image-Guided Surgery, Strasbourg, France; 3 Intuitive Surgical, Aubonne, Switzerland; 4 ICube Laboratory, Photonics Instrumentation for Health, Strasbourg, France; 5 Department of Surgery, University Hospital of Geneva, Geneva, Switzerland; 6 Medical Faculty, University of Geneva, Geneva, Switzerland

**Keywords:** Single-snapshot imaging of optical properties (SSOP), Fluorescence-based Enhanced Reality, Hyperspectral-based Enhanced Reality, Endogenous Imaging, Laser Speckle Imaging, Surgical Optomics, Colorectal anastomotic oxygenation

## Abstract

**Background:**

Adequate tissue oxygenation at the anastomotic site is a key determinant of healing after colorectal surgery. Current intraoperative imaging technologies aimed at assessing bowel perfusion present several limitations, including the need for exogenous contrast agents, limited quantification capabilities, or suboptimal integration within the surgical workflow. Single Snapshot Imaging of Optical Properties (SSOP) is a contrast-free optical imaging technology capable of quantifying tissue oxygen saturation (StO_2_) in real time. This first-in-human observational study aimed to evaluate the intraoperative safety and technical feasibility of SSOP imaging using the Trident system during left-sided colorectal resections.

**Methods:**

In this prospective, single-center feasibility trial, patients undergoing elective left-sided colorectal resection with primary anastomosis were enrolled. After bowel exteriorization, surgeons identified the intended proximal transection line based on their clinical judgement. SSOP imaging was then performed to quantify tissue oxygenation along the exposed colon segment before resection. The primary endpoints of the study were assessing the overall intraoperative safety and workflow feasibility of the Trident system. The secondary endpoint was to obtain wide-field oxygen saturation (StO_2_) status images of exteriorized human colon and studying the relationship between oxygen saturation images and a priori surgeon-determined transection lines.

**Results:**

Between September 2023 and April 2024, 10 patients were enrolled (8 males; mean age 65.5 ± 11.8 years; mean BMI 27.5 ± 4.6 kg/m^2^). SSOP imaging was successfully completed in 9 patients. The mean time required for device positioning and image acquisition was 4.45 ± 0.53 min. Surgeons rated the overall workflow as excellent or good in 100% of cases and no sterility breaches occurred. Quantitative SSOP analysis demonstrated that a priori surgeon-defined transection lines coincided with regions presenting maximal oxygenation in one of the nine cases; in all other cases, transection lines were located well within perfused tissue or proximal to oxygenation transition zones identified by SSOP. No anastomotic leaks were observed.

**Conclusions:**

This first-in-human study demonstrated that SSOP imaging using Trident is safe, feasible, and compatible with standard surgical workflow during left-sided colorectal resections. The ability to provide real-time, quantitative, contrast-free mapping of bowel oxygenation may represent a promising tool for intraoperative perfusion assessment. Larger prospective studies are needed to evaluate whether SSOP-guided surgery can improve anastomotic outcomes (NCT05973227).

**Supplementary Information:**

The online version contains supplementary material available at https://doi.org/10.1007/s00464-026-13429-6.

Anastomotic leak (AL) is the most severe complication that can occur after colorectal surgery, entailing high morbidity and mortality rates and high economic implications [1]. The incidence of AL ranges from 2 to 19% [2], with significant prevalence in left-sided colorectal resections. In practice, excess healthcare costs related to AL occurrence are estimated to be up to 9500 days of additional hospital stays and circa 28,6 million US dollars for every 1000 patients in the USA [1]. Even a 10% reduction of leakage rates would produce a substantial improvement in postoperative outcomes and a considerably positive economic impact on healthcare expenditures (approximately a two-million-dollar economy per 1000 patients undergoing left-sided colorectal resections) [1].

The main factor positively influencing anastomotic healing is an adequate oxygen supply to the anastomosis [3]. Intestinal viability is clinically evaluated by the surgeon, based on the colour of the serosa, the presence of peristalsis and the pulsation or bleeding from marginal arteries. However, clinical evaluation remains subjective regardless of clinical experience, and has only a limited value in predicting AL [4]. Additionally, if the resection has been performed in a poorly perfused area, it is quite unlikely that compensatory collateral perfusion will improve during the first 5 postoperative days [5].

Therefore, it is crucial that an anastomosis is performed in a well perfused intestinal area, and that such favourable area is properly identified intraoperatively. Optical intraoperative imaging modalities, including Fluorescence Angiography (FA) [6–9] and hyperspectral imaging (HSI) [10, 11] are promising methods to help surgeons better estimate perfusion and bowel oxygenation, respectively.

FA is an optical imaging modality that requires the systemic injection of an exogenous fluorophore (typically indocyanine green, or ICG for short) to enhance the visual perception of perfusion. Current evidence and consensus conferences suggest a potential role of FA to reduce the risk of AL in left-sided resections [8, 12]. However, FA protocols are inhomogeneous, and, in most cases, FA is still done without quantifying the fluorescent signal, which is increasingly recognized as a crucial limitation, preventing from correctly assessing actual perfusion levels [13, 14].

Hyperspectral imaging (HSI) is an endogenous optical imaging modality that can capture and quantify tissue oxygen saturation in quasi real-time by measuring light-tissue interactions (particularly reflectance) without the need of injecting a fluorescent dye. Clinical experiences with HSI technology in gastrointestinal surgery have validated its ability to accurately quantify bowel oxygenation during surgical procedures [15–23]. However, early-generation clinical his systems were mainly limited by their acquisition speed (often requiring several seconds per image) and by the fact that StO_2_ maps were displayed on a separate monitor rather than directly overlaid onto the surgical field [17]. These historical constraints are being actively addressed by advanced system designs, including lightfield imaging, workflow-integrated laparoscopic platforms, and snapshot multispectral imaging, which strive for near-real-time acquisition and augmented visualization [24].

Building upon these advancements to fully achieve video-rate, contrast-free, intraoperative optical imaging that allows for precise quantification of digestive tissue oxygenation, both in real time and at video rates, without disrupting surgical workflow, a specific technology class known as Single Snapshot of Optical Properties (SSOP) has been developed. The most accurate implementation of such technology can measure the absorption and reduced scattering properties of living tissues, allowing proper separation between sources of contrast, and thereby improving robustness in quantifying oxygenation and minimizing variability due to operating and patient-specific conditions [25–29]. A surgery-specific, real-time implementation of SSOP technology has led to the assembly of a novel contrast-free optical imaging device: the Trident System v1.0 [30–33] (Fig. 1). Trident provides accurate quantification of tissue oxygenation (StO_2_), as demonstrated in pre-clinical studies in experimental animal models of stomach, liver and bowel ischemia [34–36].

**Fig. 1:**
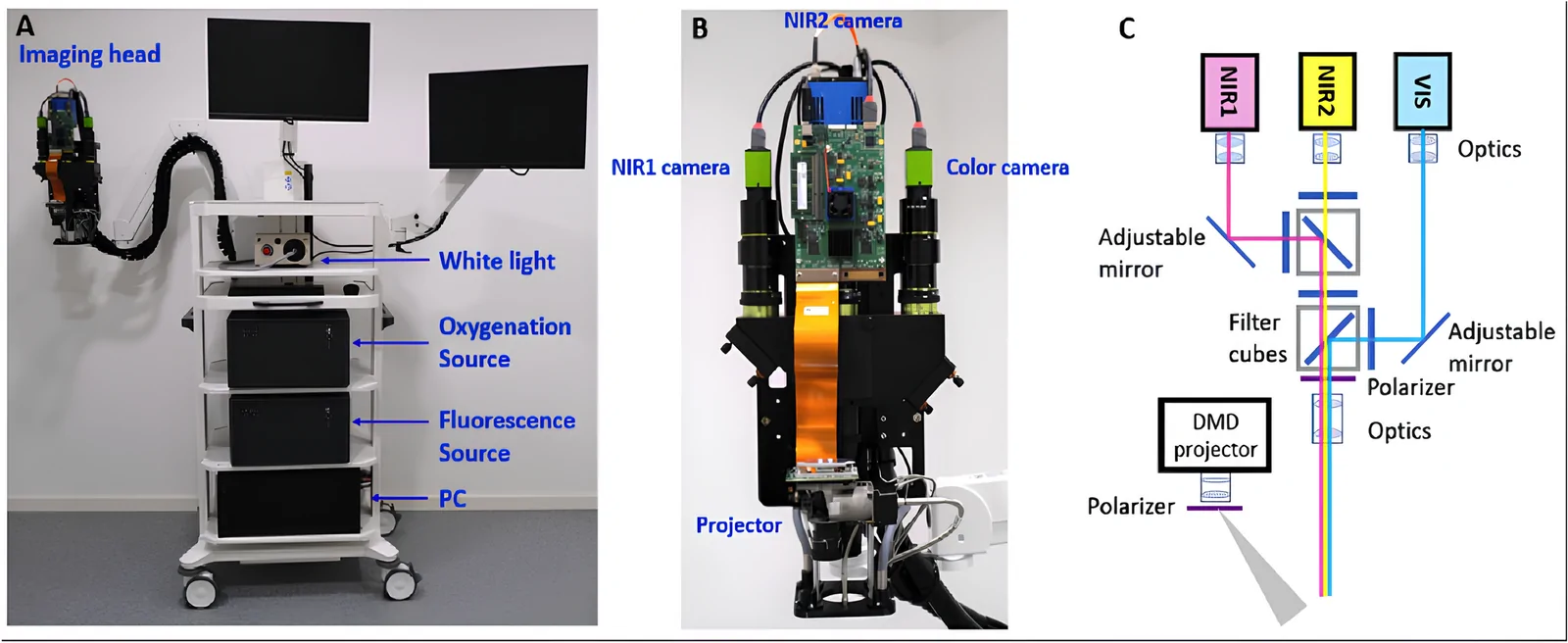
**A** The Trident System v1.0 is composed of an imaging head mounted on an articulated arm for easy positioning over any surgical scene. Its cart contains various NIR sources for oxygenation and fluorescence, a white light source, and a workstation. **B** Close-up of the imaging head, showcasing three separate optical paths for RGB, NIR1, and NIR2 imaging. A Digital Micromirror Device-based projection system is also mounted on the head, allowing for structured illumination over the entire field of view, as required in SSOP imaging. The light source is delivered to the projector via a fiber-based coupling system. An illumination ring is employed to homogeneously deliver white light illumination of the surgical field and the fluorescence excitation wavelength. **C** Schematic depicting each of the optical paths and sensors, namely the VIS camera, a standard RGB camera (JAI GO-5000C-USB) for imaging within the visible range (400–650 nm); the NIR1 camera (JAI GO-5000 M-USB, JAI, Japan) for capturing a first near-infrared channel (centered around 665 nm); and the NIR2 camera (pco.edge 4.2, PCO AG, Germany) selected to receive a second near-infrared channel (700–900 nm). This trident-shaped architecture allows for direct co-registration of the three imaging channels. A pair of filtering cubes are used to isolate the NIR1 and NIR2 channels, and a set of mirrors are employed to align the field of view of the three cameras

The aim of this first-in-human, observational trial was to clinically evaluate an SSOP system in a clinical setting, and to verify the technical feasibility and safety of the Trident system during left-sided colorectal surgeries.

## Patients and methods

### Patients and ethics

This prospective, non-interventional, feasibility and safety trial was run at the Surgical Department at Strasbourg University Hospital. It was approved by the French National Ethical Committee (Comité de Protection des Personnes; CPP) and registered under reference No. 2022-A00299-34. The trial was registered on ClinicalTrials.gov with reference code NCT05973227 and was titled “InTRaoperative Imaging DEvice Based on endogeNous opTical Properties to Evaluate Bowel Oxygenation in Colorectal Resections (TRIDENT)”.

#### Inclusion criteria

(1) patients scheduled for a left-sided colorectal resection, irrespective of the surgical approach (open, laparoscopic or robotic surgery) because of sigmoid diverticular disease, colon or rectal cancer with simultaneous restoration of digestive continuity by colorectal or coloanal anastomosis; (2) age > 18 years old; (3) ability to understand the information related to the study protocol; (4) written informed consent signed; and (5) affiliation to the French Social Security System.

#### Exclusion criteria

(1) pregnancy or breastfeeding; (2) contraindications to anesthesia; (3) inability to provide a written informed consent; (4) inclusion in a different ongoing clinical trial; (5) emergency procedure; (6) abdomino-perineal resection; (7) Hartmann procedure; and (8) whether the subject belonged to any vulnerable population sectors (e.g., prisoners, mentally disabled).

Written informed consent was obtained for all the included patients.

### Single-snapshot of optical properties

The Trident system was designed to produce wide-field, quantitative image tissue oxygenation (StO_2_) maps during open surgical procedures in a contrast-free and contactless fashion. Trident is based on Single Snapshot Imaging of Optical Properties (SSOP) technology, which in turn is a variant of Spatial Frequency Domain Imaging (SFDI). SFDI’s ability to image and quantify tissue oxygenation has been demonstrated in the past at both preclinical and clinical levels [25–29]. However, SFDI requires acquiring several frames of a given scene with multiple sinusoidal patterns and cannot perform real-time imaging; SSOP, in turn, allows for video-rate acquisition, requiring only one pattern to be captured at a time, at the expense of image quality [30–33]. So far, SSOP has never been tested in humans. The present study represents the first attempt to obtain clinical experience with this new technology.

### Study endpoints and sample size calculation

This was a safety trial. The primary endpoint was the evaluation of the workflow for the SSOP imaging device in the operating room, with a focus on image acquisition speed and overall safety, including but not limited to its potential for breaching sterility.

The rationale for choosing this primary endpoint was that, to the best of the authors’ knowledge, SSOP imaging had never been previously validated in humans nor in real clinical conditions. Despite the fact that the prototype has been largely validated in vivo in animal models with promising results in terms of accuracy of the oxygenation measurements [34], its direct translation to human cases needed to be, nonetheless, properly evaluated.

The secondary endpoint was the measurement of oxygen saturation levels (StO_2_%) throughout the entire length of the exteriorized colon before resection using Trident. The level of oxygenation (StO_2_%) provided by the system at the transection line chosen by the surgeon (which was based strictly on direct clinical evaluation and established prior to SSOP imaging) was included in each image and therefore properly recorded.

The rationale behind choosing this secondary endpoint was that the authors were interested in obtaining wide-field quantitative data for oxygenation status in human cases, as well as assessing the degree of agreement/disagreement between the surgeon and the imaging method when identifying optimally oxygenated areas within the field of view.

Considering the scope of this observational, non-interventional, safety and feasibility study, no formal hypothesis was available to estimate the population size that would be needed to obtain relevant and scientifically sound results. Based on our preclinical experience with Trident and on previous clinical translation experience with other devices of similar characteristics, we considered that a data set consisting of 10 patients would be sufficient to assess any relevant workflow or safety issues.

Therefore, we planned to enroll 12 patients in this pilot study and aimed to obtain a *complete dataset* of 10 patients. A dataset would be considered *complete* if the following conditions were met:

- Images/videos (including the exteriorized colon at the proximal resection site) were captured with Trident before completing resection and specimen removal.
- Responses to the evaluation form were registered from at least 2 distinct operators.

### Intraoperative collected parameters and post-operative questionnaire

Both quantitative and qualitative data were collected and analyzed in this study. Quantitative intraoperative parameters included the type of surgical approach, the time required for device positioning and SSOP image acquisition, and the total time elapsed between the moment when ischemia started and when images were acquired (*ischemia time*). A detailed questionnaire **(**Table 1**)** was used to collect qualitative information regarding intraoperative workflow and system usability. The evaluated items included overall workflow, ease of draping, overall duration of the imaging procedure, field of view, working distance, sterility profile, white-light image quality (overall appearance, sharpness, contrast, brightness and illumination, clarity/absence of artifacts, and image stability), ease and rapidity of cart positioning, ease and rapidity of imaging-head positioning, and rapidity of image acquisition. Each item was scored using a Likert scale ranging from Excellent (4) to Poor (1), with an additional N/A option.. The processed SSOP oxygenation maps were not presented to the surgeons for intraoperative assessment; therefore, the questionnaire did not include a qualitative evaluation of SSOP image quality. Additionally, their surgical experience as well as surgeons’ surgical experience with optical imaging systems was recorded. The questionnaire was completed immediately after surgery; postoperative clinical outcome data were recorded.

**Table 1:** Surgical questionnaire to evaluate workflow and performance

Questions	Likert grading					If fair, poor or N/A please, explain why…
#1. SSOP TRIDENT DEVICE AS A WHOLE. How do you rate the system with respect to suitability for intraoperative clinical use in open surgery regarding…	Excellent (4)	Good (3)	Fair (2)	Poor (1)	N/A	
Overall workflow (is it practical to use)						
Ease of draping						
Overall duration of imaging procedure						
Field of view						
Working distance						
Sterility profile						
#2. White light imaging. how do you rate the state and quality of the trident system with respect to suitability for intraoperative clinical use regarding…	Excellent (4)	Good (3)	Fair (2)	Poor (1)	N/A	
Overall image appearance						
Sharpness						
Contrast						
Brightness & illumination						
Clarity (no artifacts)						
Stability of the images						
#3. SSOP oxygenation imaging. How do you rate the system with respect to ease of operation for image acquisition regarding…	Excellent (4)	Good (3)	Fair (2)	Poor (1)	N/A	
Ease and rapidity of cart positioning						
Ease and rapidity of imaging head positioning						
Rapidity of images acquisition						

### Surgical procedure(s)

**Fig. 2:**
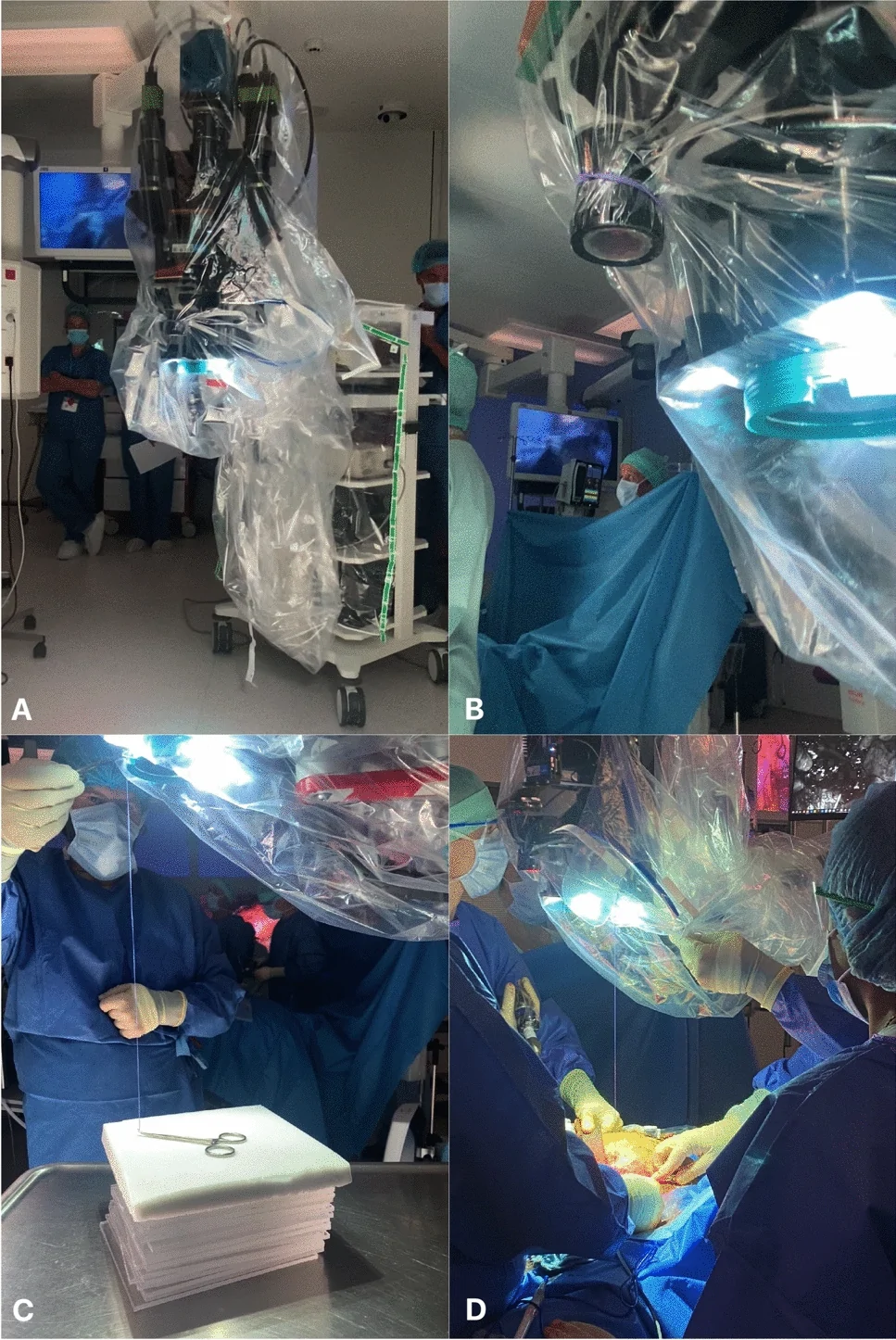
Images of Trident in the operating room. **A** Draped system ready for imaging. The imaging head, its articulated arm and the anterior aspect of the main cart are properly isolated from the surgical field. **B** Close-up of the imaging head, showing the rigid interface for the main camera viewport (in green), as well as the use of elastic rings to keep the drape in tension at the projection end, avoiding distortions of the projected pattern. **C** Working distance from the target anatomy is roughly estimated with a 40-cm-long, sterile elastic band that is also **D** used intraoperatively to position the camera at the regions of interest marked by the surgeon (i.e., the transection line)

All consecutive patients scheduled for a left-sided colonic or rectal resection with colorectal or coloanal anastomoses were assessed for enrollment with the aim to enroll a total of 12 patients for the study. During surgical field preparation, the scrub nurse, who had received dedicated training in the draping procedure, covered the SSOP system imaging head, the articulated arm and the anterior aspect of the main cart with sterile draping, which included a rigid interface in front of the main camera viewport. An elastic ring from the sterile echo kit was also used to secure the section of the drape covering the projector head, keeping it in tension and therefore avoiding any distortions of the projected pattern on the field of view that could have been potentially caused by the drape **(**Fig. 2 A, B).

All surgical procedures were performed by senior staff surgeons (A.L. and G.P.). The system itself was operated by a dedicated non-sterile operator (S.K.), who had been specifically trained in the use of the imaging system. The operating surgeons (A.L., G.P.) were not involved in operating the system or initiating the image acquisition; their interaction with the device was limited to guiding the positioning of the imaging head over the target anatomy and indicating the appropriate timing for image acquisition.

Sigmoid resections for benign disease were performed with a standard dissection of the mesocolon close to the bowel using a LigaSure™ vessel-sealing system (Valleylab, Boulder, Colorado, United States) and without ligation of the inferior mesenteric artery at its origin. A splenic flexure takedown was performed only when necessary. A protective ileostomy was made whenever needed according to surgeons’ indication. Left colectomies and low anterior resections for cancer involved oncologic dissection using a medial-to-lateral approach and division of mesenteric vessels. A splenic flexure takedown was routinely performed in rectal cancer patients.

After the dissection phase, the distal bowel transection and exteriorization were achieved through a Pfannenstiel incision; then, the surgeon marked the intended proximal resection line on the bowel with metallic clips or with a surgical marker, based on direct clinical evaluation. At this point, the Trident system was brought near the operating table. Under the guidance of the operating surgeon, the imaging head was placed at a fixed distance of approximately 40 cm from the target anatomy, roughly estimated with the help of a 40 cm-long sterile elastic band (Figs. 2C and D). Once the surgeon confirmed the appropriate positioning and timing for imaging, SSOP acquisition was initiated by the dedicated non-sterile operator through the system interface.

After SSOP imaging, the proximal surgical resection was performed strictly based on the previously marked clinical decision. A mechanical end-to-end or side-to-end anastomosis was performed according to surgeons’ preference and was systematically tested with an air-leak test via flexible endoscopy at the end of the procedure.

### Statistics

Only basic descriptive statistics on recorded data for both primary and secondary endpoints were performed. We enumerate them below.

Concerning the primary endpoint of the study, we performed descriptive statistics about the Likert-scale scores provided in questions (a) through (e) indicated in the previous section.

To address the secondary endpoint of the study and to carefully evaluate the decay in oxygenation levels between the proximal and distal ends of each segment, a semi-automated quantitative analysis was performed at post-processing, with the objective of standardizing and aligning all patients length-wise with respect to the transection line decided by the surgeon, at a longitudinal resolution that would be appropriate for the aforementioned workflow (i.e., centimeters).

**Fig. 3:**
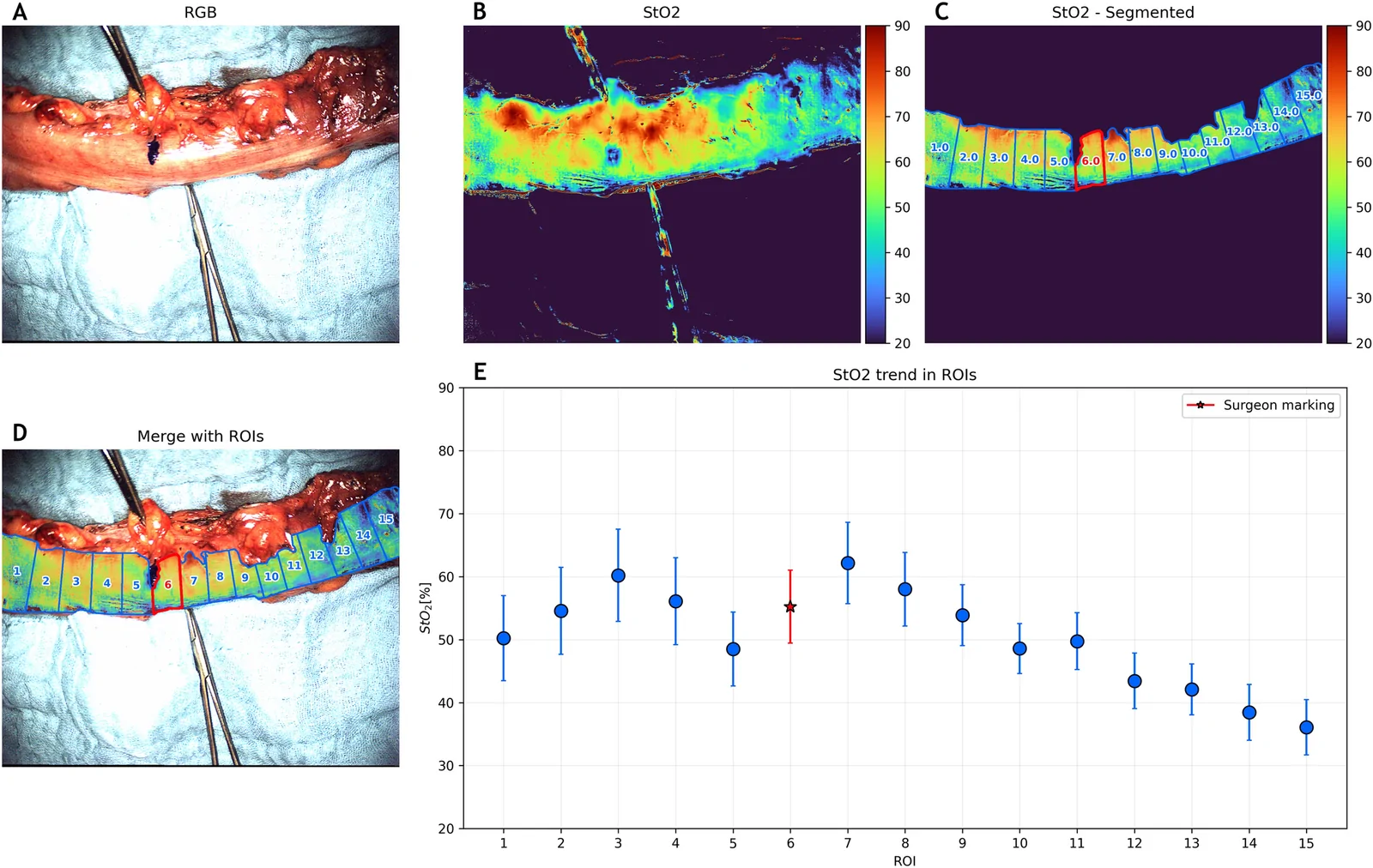
Detailed post-processing analysis of SSOP oxygenation images from a single patient. From left to right, top to bottom: **A** surgical view in RGB/white light; **B** SSOP-based StO_2_ map; **C** valid one-centimeter-long regions of interest of the exposed serosa calculated via k-means; **D** surgical view including overlay of the partitioned StO2 map; **E** unwrapped StO_2_ mean and standard deviation per region of interest

The procedure was as follows. For each image and patient, two binary masks were manually generated. A first mask (the *coarse mask)* represented the overall trajectory of the exposed colonic segment as observed across the field of view, and allowed us to separate specimen from background (Fig. 3B); another mask (the *exposed colon mask*) was created to exclusively select regions of exposed colonic tissue (i.e., serosa), and to reject adipose tissue, blood, char, and similar artifacts that would bias our statistics in downstream tasks (Fig. 3C).

**Fig. 4:**
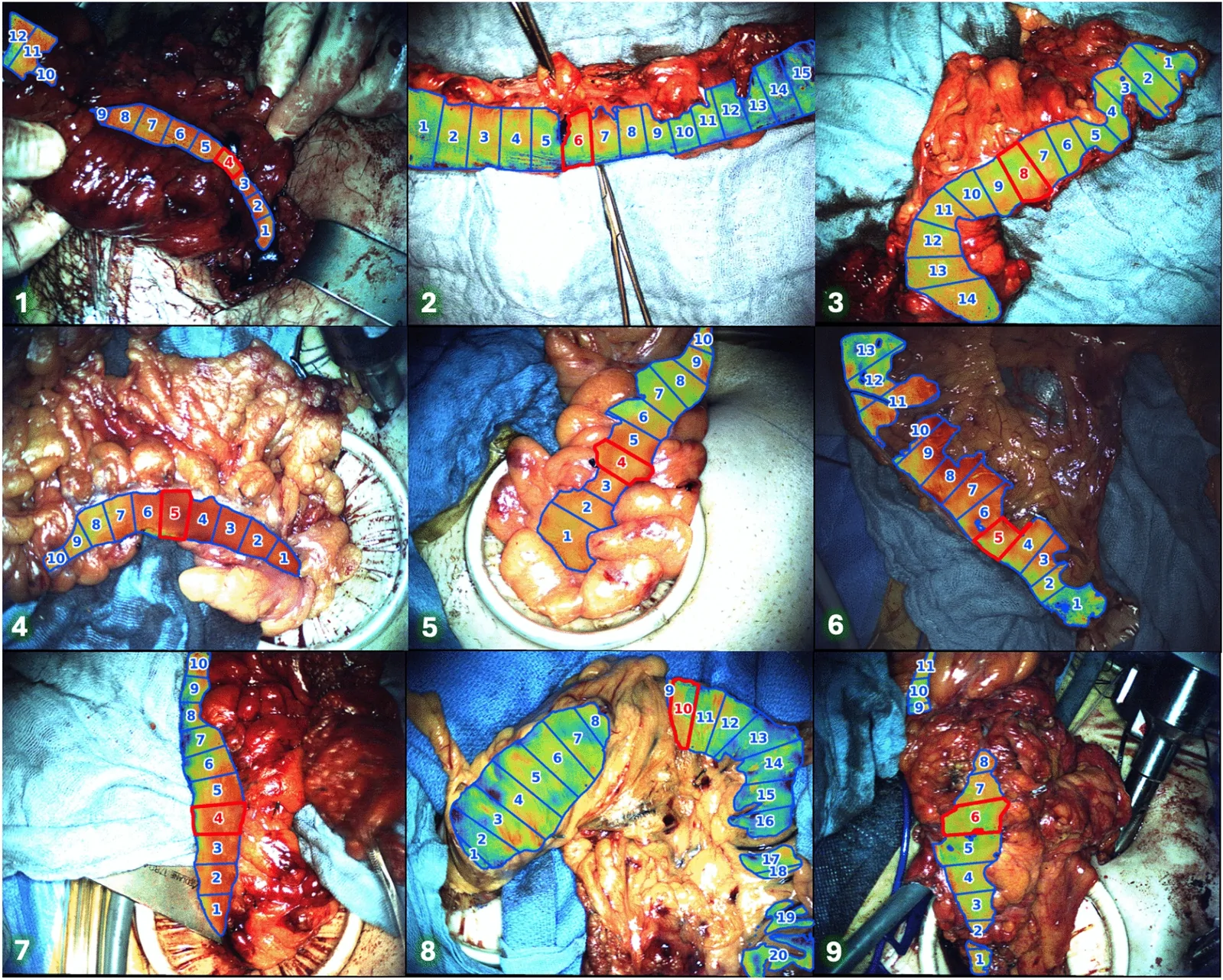
Intraoperative SSOP images of exteriorized colon. We present the main surgical scene and overlay their associated oxygen saturation maps, as well as the partitioning of the serosa into one-centimeter regions of interest (ROI) for each patient. The transection line for each case (decided by the surgeon under white light during the intervention and without SSOP) is highlighted in red. Numbers index all ROIs from the proximal to the distal end of each exteriorized colon segment

**Fig. 5:**
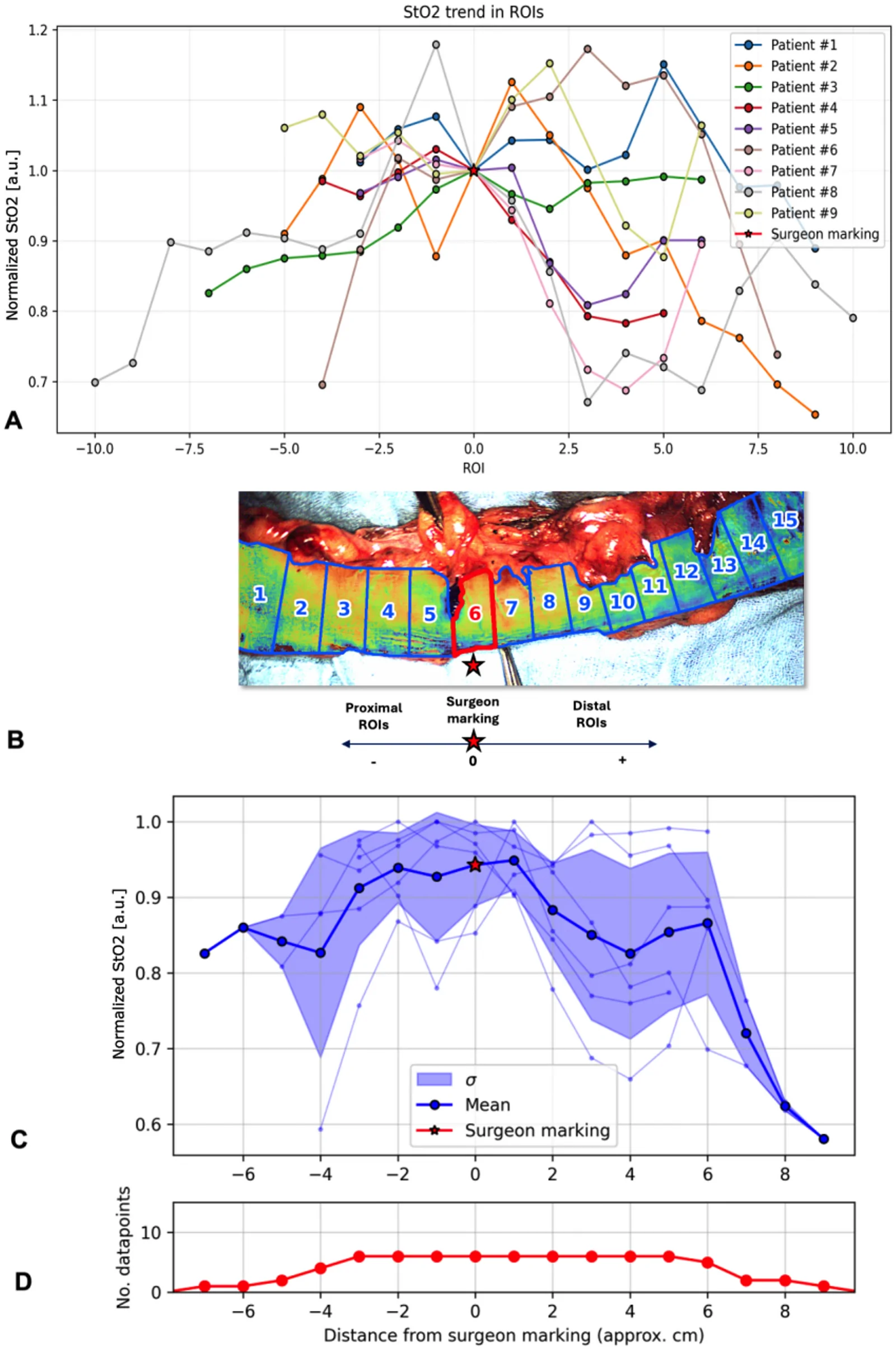
**A** Quantitative analysis of oxygen saturation (StO_2_) values for all patients, aligned longitudinally with respect to the region of interest closest to the transection line indicated by the surgeon; **B** Surgical view with overlaid SSOP-based StO_2_ map and regions of interest (ROIs); the transection line selected by the surgeon is shown in red, and ROIs are numbered in proximal to distal order. **C** Inter-patient mean and standard deviation of StO_2_ values normalized with respect to the region of interest closest to the transection line. **D** Number of datapoints per region of interest (i.e., the number of ROIs is not the same for all patients due to interpatient variability)

These masks were sufficient to systematically describe centimeter-long subsections of the exposed colon, as long as two additional algorithms were applied. Both algorithms were performed independently for each image and patient. The first task was to perform a *morphological skeletonization* of the coarse mask, resulting in a trajectory that roughly approximated the centerline of the colon segment visible in a given image. Since this was not a perfect procedure—because manual labels are inevitably non-convex—secondary branches for all skeletons were manually pruned, and only the main trajectory from proximal to distal was preserved. Immediately afterwards, we ran *k*-means clustering on this skeletonized mask; doing so resulted in discrete positions at roughly constant distances from one another, all positioned along the main centerline of the visible organ. The choice for the number of clusters was performed iteratively for each trajectory; knowing the working distance and the field of view, we were able to roughly establish the pixel resolution and, with that information in mind, we gradually increased the number of centroids in the trajectory until the nearest-neighbor distance for every centroid reached one centimeter or less (±0.1 cm). At last, the obtained centroids were used to split the exposed serosa (defined by the *exposed colon mask*) into equally distanced regions of interest along the trajectory of the colon **(**Fig. 3C). The oxygen saturation was then averaged for each of these regions, and the resulting values were unwrapped from proximal to distal and plotted on the real line, following the order observed in the original trajectory. The region closest to the transection line—which was manually indicated by the surgeon, who pointed at the location with an instrument in nearly all images—was annotated for each patient; this allowed us to longitudinally align all organs at the transection line, and evaluate the differences in oxygen saturation on the proximal and distal sides of this decision boundary at regularly separated locations (Fig. 3D). Finally, we evaluated interpatient variability and population size for each of the regions of interest once all specimens were aligned (Figs. 4 and 5).

To facilitate the interpretation of oxygenation gradients along the exteriorized colon, StO₂ values were normalized for each patient with respect to the maximum SSOP value detected within the analyzed field of view, which was therefore set to 100%. This normalization allowed the comparison of oxygenation profiles across patients independently from interpatient variability in absolute StO₂ measurements. Based on this normalized distribution, a patient-specific threshold for adequate perfusion (cutoff value) was calculated as the mean ± standard deviation of the normalized StO₂ values measured from the proximal extremity of the colon segment up to the region of interest corresponding to the maximum oxygenation value. This range was considered representative of physiologically well-perfused tissue. Regions presenting StO₂ values falling within this interval were therefore classified as adequately perfused, while regions with values below this threshold were interpreted as belonging to the transition toward less perfused tissue. This approach allowed us to objectively identify the transition between well-oxygenated and progressively hypoperfused bowel segments and to compare this point with the transection line chosen intraoperatively by the surgeon (Table 2).

**Table 2:**
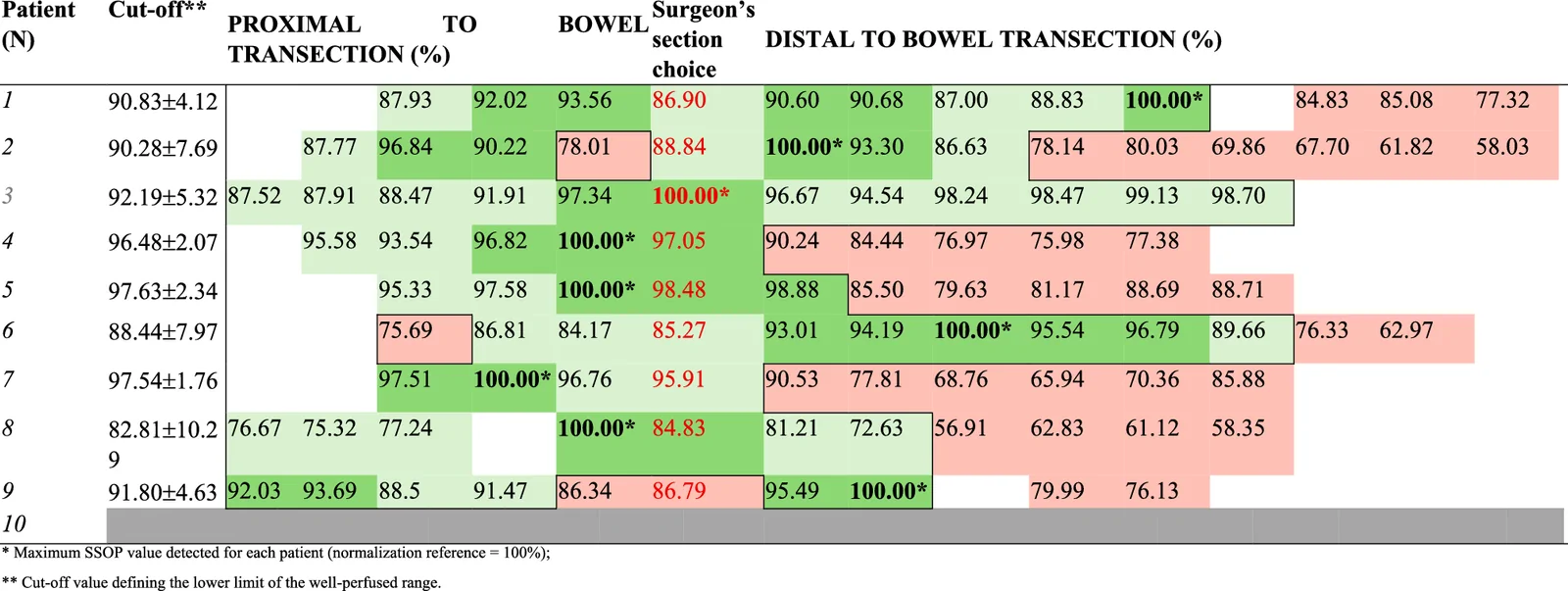
Normalized SSOP StO₂ values measured along the exteriorized colon

^* Maximum SSOP value detected for each patient (normalization reference = 100%);^

^** Cut−off value defining the lower limit of the well−perfused range.^

For each patient, oxygen saturation values were calculated for sequential one-centimeter regions of interest (ROIs) along the exposed colon segment. Oxygen saturation values were normalized with respect to the maximum value observed in each patient. The table reports the normalized oxygenation values proximal and distal to the transection line chosen by the surgeon. The cut-off value for adequate perfusion was calculated for each patient as the mean ± standard deviation of the normalized StO₂ values from the proximal extremity of the colon to the ROI showing the maximum oxygenation value, representing the range of physiologically well-perfused tissue.

## Results

From September 2023 to April 2024, thirteen patients were assessed for enrollment. Two patients did not meet the inclusion criteria, and one withdrew their participation before the surgery; in the end, a total of 10 patients (8 male, 2 female; mean age: 65.5±11.8; mean BMI: 27.5±4.6 m/Kg^2^) were enrolled in the protocol. Two senior staff surgeons (A.L. and G.P.) performed all the surgeries. Clinicopathologic characteristics of the patient population are summarized in Table 1.

In terms of timing, the mean total time required to position the device and to acquire SSOP images was 4,45 ± 0,53 min, and the mean ischemia time before imaging was 160,89 ± 144,10 min. However, it ranged widely from 74 to 536 min (median: 116 min). The 536-min outlier corresponded to a technically complex robotic ultra-low anterior resection with Total Mesorectal Excision in an obese patient presenting a very high splenic flexure and severe adhesions following multiple previous abdominal procedures, which substantially prolonged the interval before SSOP acquisition.

All patients underwent minimally invasive surgeries (five were laparoscopic, five robotic). Only one conversion from laparoscopic to open surgery was required, due to a frozen pelvis secondary to complicated stenotic diverticular disease presenting with sigmoido-vesical, entero-colic, and colo-colic fistulas.

### Surgeon questionnaires

**Fig. 6:**
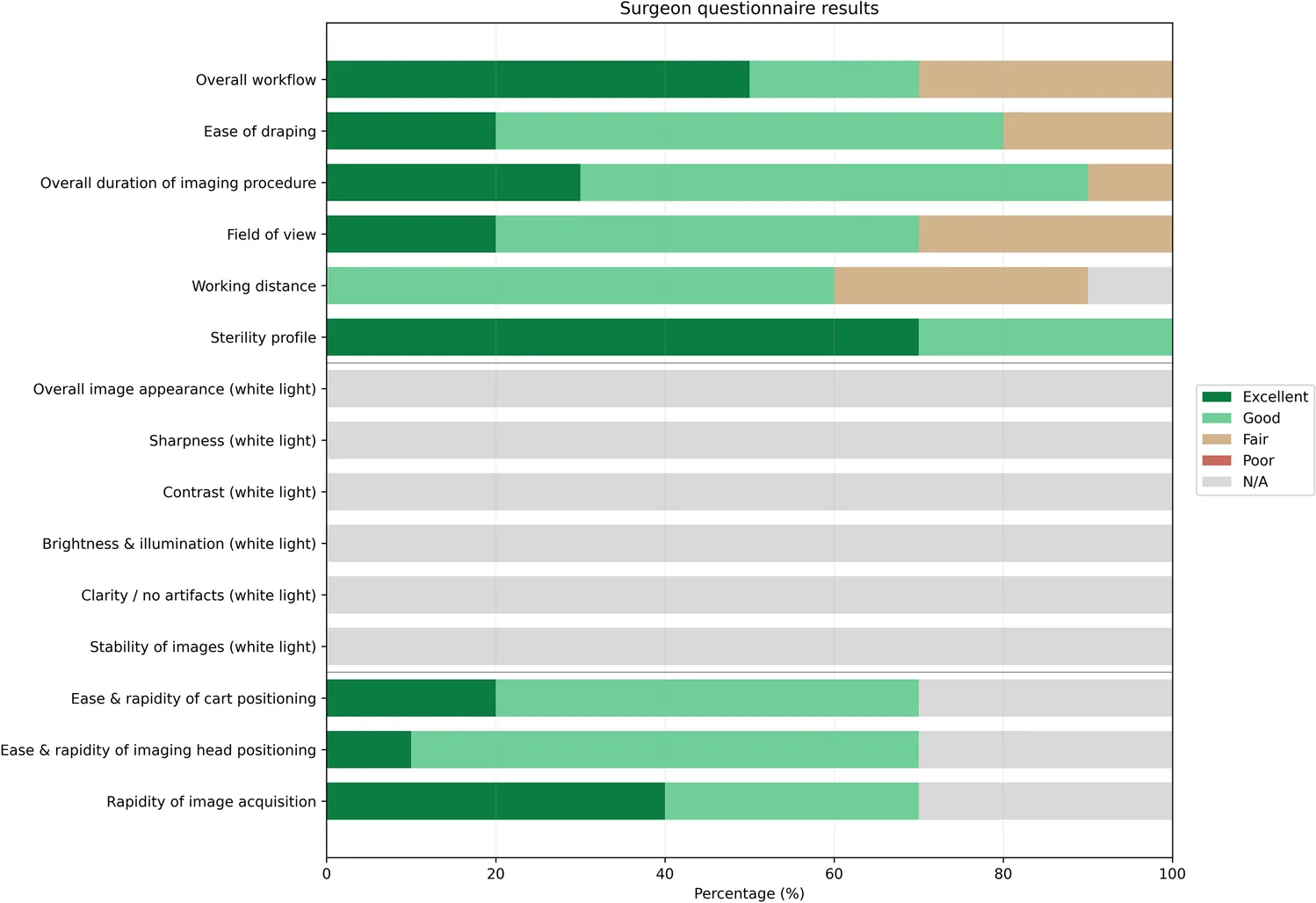
Surgeon questionnaire results

Both operating surgeons had previous experience with intraoperative optical imaging, including fluorescence angiography and hyperspectral imaging, through their participation in previous clinical and/or experimental studies. However, neither surgeon had prior experience operating the Trident system, as the device operation and image acquisition in the present study were performed exclusively by the dedicated trained non-sterile operator. Questionnaire results are summarized in Fig. 6. Overall workflow was rated excellent in 50%, good in 20%, and fair in 30% of cases. Ease of draping was rated excellent or good in 80% of cases, while the overall duration of the imaging procedure was rated excellent or good in 90%. Field of view was rated excellent or good in 70% of cases and fair in 30%. Working distance was rated good in 60, fair in 30, and N/A in 10%, whereas the sterility profile was rated excellent or good in all cases. All white-light image-quality items were rated N/A. Ease and rapidity of cart positioning, imaging-head positioning, and image acquisition were rated excellent or good in 70% of cases, with 30% N/A responses for each. No poor ratings were reported for any evaluated item.

### Intraoperative, real-time SSOP analysis

SSOP imaging was achieved in all cases except for one patient, where a technical problem related to the optical system resulted in imaging system failure that was only discovered during data post-processing, preventing a successful acquisition. The full dataset therefore consists of SSOP images of 9 patients (5 sigmoidectomies for diverticulitis, 2 oncologic sigmoidectomies, 1 oncologic left colectomy and 2 low anterior resections for cancer) (Table 2 and 3).

**Table 3:** Clinicopathologic characteristics of the populations

	Gender	Age (years)	BMI (Kg/m^2^)	Pathology	Intervention	Surgical approach	Conversion	Vascular ligation	Anastomotic type	Protective stoma	Neoadjuvant treatment	Anastomotic leak	Reoperation	Length of hospital stay (days)
Patient 1	M	41	33,48	Diverticulitis	Sigmoidectomy	laparoscopic	No	Low-tie	Side-to-end	No	No	No	No	4
Patient 2	M	66	21,72	Adenocarcinoma of the rectum	Anterior rectal resection	laparoscopic	No	High-tie	Side-to-end	No	Yes	No	No	4
Patient 3	M	77	22,65	Diverticulitis with colovescical, colocolic and enterocolic fistula	Sigmoidectomy with segmental small bowel resection, lateral stoma closure	laparoscopic	Yes	Low-tie	End-to-end	Yes	No	No	Yes	41
Patient 4	M	64	27,68	Diverticulitis	Sigmoidectomy	robotic	No	Low-tie	End-to-end	No	No	No	No	4
Patient 5	M	76	32,65	Diverticulitis	Sigmoidectomy	robotic	No	Low-tie	End-to-end	No	No	No	No	4
Patient 6	F	79	23,44	Adenocarcinoma of the descending colon	Left colectomy	laparoscopic	No	High-tie	End-to-end	No	No	No	No	5
Patient 7	M	55	33,03	Adenocarcinoma of the sigmoid colon	Sigmoidectomy	robotic	No	High-tie	End-to-end	No	No	No	No	2
Patient 8	M	58	24,91	Diverticulitis	Sigmoidectomy	laparoscopic	No	Low-tie	End-to-end	No	No	No	No	5
Patient 9	F	73	30,82	Adenocarcinoma of the rectum	Low anterior resection	robotic	No	High-tie	End-to-end	Yes	Yes	No	No	8
Patient 10	M	67	27,00	Diverticulitis	Sigmoidectomy	robotic	No	Low-tie	End-to-end	Yes	No	No	No	6

The results for SSOP are reported in Fig. 4, 5 and Table 2. Interestingly, the surgeon’s transection line coincided with the region of highest mean oxygen saturation only in one of the nine patients (11,12%). In other four patients (44,44%), the point of maximum oxygen saturation was distal to the transection line and, in the remaining four patients (44,44%), the point of maximum oxygenation was more proximal with respect to the transection line. We will refer to the first group as the *distal* cases, and to the second as the *proximal* cases, respectively. In three of the four proximal cases, the regions of interest with maximum oxygen saturation were at about 1 cm proximal to the transection line, with a fourth at about 2 cm proximal to it; in the *distal* cases, however, the peak oxygen saturation section was 1 cm distal with respect to the transection line for only one case, with the rest of the oxygenation peaks at 2, 3, and 5 cm distal to the transection line, respectively (average distance was 3.33 ± 1.53 cm). Despite there being a case in the proximal group where the maximum oxygen saturation was 2 cm proximal to the transection line, the average saturation value of the ROI corresponding to the transection line in this patient still fell well within acceptable perfusion values, as defined in the Statistics section (Table 2).

### Postoperative outcomes

No anastomosis-related complications occurred in this study; The overall mean postoperative length of stay was 8.3 days, which was heavily skewed by an extended hospitalization for Patient #3 due to complications unrelated to the study device or the anastomosis. When excluding this patient, the mean postoperative length of stay for the remaining cohort was 4.67 ± 1.66 days (Table 3).

Specifically, Patient #3, who initially underwent surgery for complicated diverticular disease with chronic sigmoido-vesical, entero-colic and colo-colic fistulae, required reoperation for a uroperitoneum resulting from bladder dome urine leak. This was secondary to the premature removal of the urinary catheter on postoperative day 2 (Clavien-Dindo grade III-b). This event lead to subsequent pulmonary and urinary complications prolonging the hospital stay to 41 days. Aside from this case, there were no other major postoperative complications (Clavien-Dindo ≥ III).

## Discussion

This first-in-human observational trial demonstrated the intraoperative safety and technical feasibility of SSOP-based oxygenation imaging using the Trident system during left-sided colorectal resections. Our results demonstrate that this system is able to provide quantitative, contrast-free assessment of bowel oxygenation (StO_2_) with negligible disruption to standard surgical workflow, requiring only a few additional minutes for device positioning and image acquisition. The successful translation of SSOP into the operating room represents a step forward in the evolution of surgical guidance, addressing the critical limitations of preceding optical imaging technologies. While the primary endpoint of this study was safety and workflow evaluation, previous preclinical results using SSOP provide clear and objective assurances on the fact that StO_2_ has likely been accurately measured with Trident in this study.

Over the past decade, some of the authors have focused on linking intraoperative optical imaging data to relevant perfusion/oxygenation biomarkers. Local capillary lactates (LCL) can act as a reliable ground truth for validating perfusion technologies because measuring them constitutes a definitive metabolic marker of tissue ischemia; they rise precisely when cellular aerobic metabolism fails due to lack of oxygen. We already introduced the concept of metabolism-guided bowel resection [37], in which we were able to validate that simple and inexpensive tools to measure capillary lactate in blood directly sampled at certain regions of interest in several organs were comparable to cutting edge, highly accurate technologies such as real-time mitochondrial respiration and metabolomics profiling.

Using this same experimental setup, we were able to prove a strong and statistically significant correlation between capillary lactate levels, inflammation scores [38] and potentially anastomotic leaks [14] with the signal provided by several optical imaging modalities, including fluorescence imaging [38–43], hyperspectral imaging [17], laser speckle [16, 22] and multispectral SSOP [34–36] in both preclinical and clinical settings [14].

Given the design of this study, SSOP was not used to guide resection; nevertheless, the validity of the oxygenation values provided by the system were supported by the existence of robust preclinical results, as previously discussed. In particular, the correlation between the StO_2_ values derived from Trident and local lactate levels was statistically significant (r = 0.78, p = 0.0009) [34]. In this sense, the exploratory analysis of SSOP-derived oxygenation maps provided interesting insights into the potential clinical role of this technology. Trident, as a standalone system, integrates the main advantages of previous optical imaging modalities while addressing several of their inherent limitations. Like HSI, it is endogenous and quantitative, avoiding the safety risks related to dye injection; uniquely, however, it operates at video-rate speeds, allowing for immediate quantification of StO_2_ without all the workflow delays inherent to HSI [36].

The primary endpoint of this trial was safety, which has been confirmed both in terms of workflow and patient risk. Our workflow analysis showed that the device was positioned and images were acquired within 4.45 ± 0.53 min, with no sterility breaches nor anastomotic leaks to be reported. Although the small sample size prevented us from establishing any significant chain of causality, our analysis of oxygen saturation information in these cases suggests that surgeons tended to select transection points located in well-perfused areas, frequently proximal to the transition zone between well-oxygenated and less-oxygenated tissue identified by SSOP, as defined by our patient-specific oxygenation threshold derived from normalized saturation values (Fig. 5 and Table 2). Such observation may partially explain the absence of anastomotic complications in this cohort, as the surgeons’ clinical judgment often resulted in resection margins placed within adequately oxygenated tissue (Table 2).

However, quantitative analysis of SSOP-based oxygen saturation images also revealed that, in several cases, regions with adequate oxygenation may have extended further distally from the site chosen by the surgeon. This finding highlights a potential added value of objective oxygenation mapping: the ability to precisely identify the transition between adequately perfused and marginally perfused bowel. From a surgical perspective, this capability may have two important implications. First, it could help prevent unnecessarily proximal resections by providing quantitative confirmation that the bowel segment immediately distal to the surgeon’s chosen transection point remains adequately oxygenated. Preserving additional bowel length may be particularly relevant in complex colorectal procedures where conduit length is a critical factor, such as low pelvic anastomoses. Second, a more accurate identification of the true transition zone between perfused and unperfused tissue may help tailor the extent of mobilization required to achieve a tension-free anastomosis, potentially reducing the need for extensive maneuvers such as routine splenic flexure mobilization. Interestingly, in our cohort, the peak SSOP oxygenation value was occasionally located several centimeters distal to the clinically chosen transection line. Whether this represents salvageable colon (highlighting the potential added value of SSOP for tissue preservation) or 'false positives' of adequate microperfusion in clinically unsafe tissue cannot be determined from this small safety cohort. While the current first-in-human pilot study establishes the feasibility of acquiring these StO2 data safely, future adequately powered clinical efficacy trials are required to establish whether SSOP-guided resection safely improves tissue preservation, and to define the exact quantitative thresholds that correlate with safe anastomotic healing. Furthermore, these larger cohorts will allow for subgroup analyses to investigate how surgical variables, such as the choice between high-tie and low-tie vascular ligation, affect microperfusion at the anastomotic site.

In this regard, SSOP imaging could serve not only as a safety tool for identifying poorly perfused segments, but also as a precision guidance system capable of optimizing the balance between oncologic resection, vascular adequacy, and conduit preservation. While these considerations and ideas remain strictly hypothetical for now, they illustrate the potential of quantitative endogenous imaging technologies to refine intraoperative decision making beyond what can be achieved through visual assessment alone.

The study presented in this manuscript is limited by its observational nature and small sample size (nine patients). The heterogeneity of ischemic times and the lack of a control group both prevent us from drawing robust conclusions on the clinical utility of SSOP to prevent anastomotic complications. Nevertheless, the successful demonstration of safety and technical feasibility in this pilot trial provides the rationale for the next step: a prospective, multicenter interventional trial aimed at evaluating the impact of SSOP-guided surgery on anastomotic leak prevention.

## Conclusions

This first-in-human pilot study confirms that Trident—an SSOP-based imaging system—is safe, feasible, and compatible with standard surgical workflow during left-sided colorectal resections. While this observational trial was not designed to assess clinical outcomes, the ability to objectively visualize tissue metabolism in real time and without contrast paves the way towards proper clinical validation of the technology. Future large-scale, interventional trials are a necessary next step to validate whether SSOP-guided resection can effectively reduce the incidence of anastomotic leakage and improve patient outcomes.

## Supplementary Information

Below is the link to the electronic supplementary material.

Supplementary file1 (DOCX 240 KB)

## Data Availability

The authors are grateful to Armelle Takeda, Marion Chahi, Kahina Saichi and Elsa Malet, clinical research assistants, for their assistance with data collection and study management.
