# Supplementary material for: TRIDENT: inTRaoperative Imaging DEvice based on endogeNous opTical properties to evaluate bowel oxygenation in left-sided colorectal resections. Observational first-in-human pilot safety trial
Source: Surg Endosc. 2026 Oct 12;40(11):10181–95. doi: 10.1007/s00464-026-13429-6 (PMC13755004; doi:10.1007/s00464-026-13429-6)
Supplement: Supplementary file 1 [file 464_2026_13429_MOESM1_ESM.docx]

**Supplementary Figure 1. Absolute (non-normalized) tissue oxygenation (StO2) values aligned to the clinical transection line.** The top panel displays the absolute StO2 values (%) across the cohort, anchored spatially at the surgeon's marking (0 cm, red star). Absolute values generally ranged from approximately 40% to 85%, depending on the distance from the transection line. The bottom panel illustrates the number of available data points at each spatial interval across the cohort. As demonstrated in this non-normalized data, there is significant inter-sample variability in mean baseline oxygenation among patients. While the relative proximal-to-distal decay in oxygenation is the most consistent physiological finding, this critical gradient is heavily obscured by differing patient baselines when plotting only absolute values. Therefore, normalizing each curve to its maximum oxygenation value provides a much cleaner, standardized representation of the relative oxygenation falloff across the cohort.
